# High throughput automated colorimetric method for the screening of l-lactic acid producing microorganisms

**DOI:** 10.1016/j.mex.2014.10.001

**Published:** 2014-10-16

**Authors:** Nadège Liaud, David Navarro, Nicolas Vidal, Jean-Claude Sigoillot, Sana Raouche

**Affiliations:** a INRA, UMR1163 Biotechnology and Biodiversity of Fungi, F-13288 Marseille, France; b Aix Marseille Université, UMR1163 Biotechnology and Biodiversity of Fungi, F-13288 Marseille, France; c ARD, Agro-Industry Research and Development, F-51100 Pômacle, France; d Yelen, 10 boulevard Tempête, F-13820 Ensues la redonne, France

**Keywords:** ABTS, 2,2′-azino-di-[3-ethylbenzthiazoine-sulfonate], HRP, horseradish peroxidase, LLOD, lower limit of detection, LLOQ, lower limit of quantification, LOD, l-lactate oxidase, LA, l-lactate concentration, Automated screening for lactic acid production, l-Lactic acid, Screening, Automated assays, Fungi, Bacteria, Colorimetric, l-Lactate oxidase

## Abstract

Lactic acid is a valuable and fully degradable organic acid with promising applications in poly-lactic acid production (Taskila S and Ojamo, 2013 [1]). Despite their efficiency, the cost of the current lactic acid bio-processes is still an obstacle to this application (Miller et al., 2011 [2]). To ameliorate lactic acid producing strains, researchers are using mutations and metabolic engineering techniques, as well as medium optimization. All these studies necessitate a good and high throughput screening method. Currently, researchers mostly use HPLC methods which often necessitate sample preparation, are not stereospecific and do not allow high throughput. To help optimizing l-lactic acid production, we developed a high throughput colorimetric method inspired by the blood l-lactic acid detection method used for diagnosis (Lin et al., 1999 [3]).

• Two sequential enzymatic reactions using l-lactate oxidase, peroxidase and ABTS (2,2′-azino-di-[3-ethylbenzthiazoine-sulfonate]), a chromogenic peroxidase substrate, are used to quantify l-lactate between 13.8 and 90 mg/l.
• The accuracy of the method was ascertained before automation.
• The method was successfully applied for the direct determination of l-lactate content in fungal culture supernatants.

## Method details

Lactic acid is the building block for Poly-Lactic Acid (PLA), a biodegradable polymer which could replace petroleum-based plastics. With the environmental urge for ecofriendly products, the demand for this biomaterial increases the demand for lactic acid, and the market is expected to reach 1 million tons by 2020 [1]. Lactic acid bacteria are well known to secrete large amounts of L and/or D-lactic acid as a product of carbohydrate metabolism [1]. However, production costs of lactic acid using LAB are still too high restricting PLA use as a convenient product [2]. Overall production costs can be decreased by improving production step using (i) more efficient, either natural or genetically modified, microorganisms, (ii) optimizing culture conditions, e.g. low-nutrient medium with low-cost carbon source, low pH etc. For this type of studies, a fast and high throughput screening method for lactic acid production would be useful.

The colorimetric detection of l-lactate uses two sequential oxido-reduction reactions. The first one is the conversion of l-lactate and O_2_ into pyruvate and H_2_O_2_, catalyzed by the stereospecific l-lactate oxidase (LOD). In the second reaction, catalyzed by the horseradish peroxidase (HRP), the chromogenic substrate ABTS (2,2′-azino-di-[3-ethylbenzthiazoine-sulfonate]) is oxidized by the H_2_O_2_ produced in first reaction. The oxidized ABTS absorbs at 420 nm and dyes the reaction mixture in green. The ABTS oxidation is proportional to l-lactate concentration which can be calculated from a standard curve of known l-lactate concentration. The method was adapted from l-lactic acid detection methods used in diagnosis [3]. For that purpose, the reaction parameters: optimal enzyme/substrate ratios, incubation time, detection and quantification lower limits were determined. The automated assay was then performed by measuring the accuracy and the repeatability of the results obtained using a pipetting robot. Finally, to prove the practicality and accuracy of the microplate method, the assay was tested with fungal culture supernatants and the method was compared with HPLC using a method previously used for organic acid determination [4]. This two-steps quantification method can be performed at room temperature, directly on diluted supernatants. Considering measurements in duplicates with one calibration curve per plate, 80 samples could be analyzed in 1 h and the robot could potentially run analysis 24 h per day (i.e. 1920 samples per day). As a comparison, more than 3 days would be necessary to run 80 samples with a classical HPLC method.

### Preparation of the reaction mixture

The stereospecific LOD (50 U/ml, SORACHIM SA) and HRP (150 U/ml, Sigma–Aldrich) stock solutions were prepared from powders diluted in phosphate reagent buffer 0.1 M, pH 6. ABTS (5 mM, Sigma–Aldrich) and l-lactate (5 g/l, Sigma–Aldrich) stock solutions were prepared by dissolving the reagent powder in deionized water. These stock solutions were stored at 4 °C before use.

### Determination of the enzyme ratios and incubation time

**Fig. 1:**
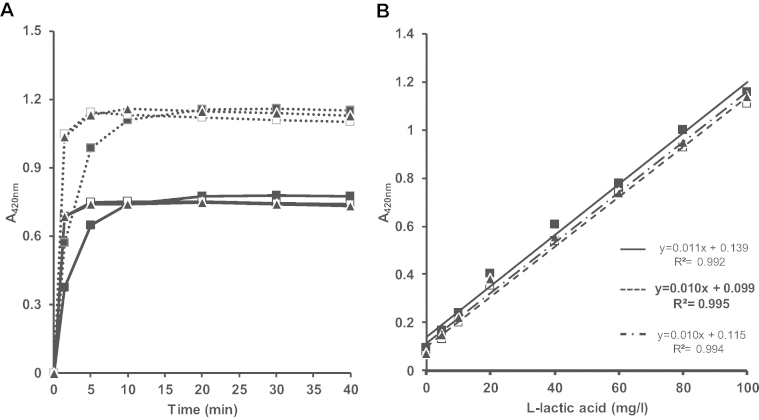
Kinetics and linear regression for l-lactate quantification by colorimetric microplate method. (A) Kinetics of the reaction obtained with mixes containing 0.25 (■), 1.5 (□) or 2.5 (▴) U/ml of LOD; at two standard l-lactate concentrations: [LA] = 60 mg/l (**—**) and 100 mg/l (**–** **–** **–**). (B) Standard curves obtained after 30 min incubation with mixes containing 0.25 (■), 1.5 (□) or 2.5 (▴) U/ml of LOD. The linear regressions and equations obtained for each LOD concentration are shown in the figure: 0.25 (**—**), 1.5 (**–** **–** **–**) or 2.5 (**–·–**) U/ml of LOD.

The first step was to determine the best ratio between the two enzymes and the end point time of the reactions. In manual assays, the HRP final concentration was fixed to 1.5 U/ml while three LOD final concentrations were assayed: 0.25, 1.5 and 2.5 U/ml. The l-lactate standards, 0, 5, 10, 20, 40, 60, 80, and 100 mg/l, were prepared with proper dilutions of the stock solution. The reaction mixture (final volume of 200 μl) contained 20 μl of 1 M phosphate reagent buffer pH 6, 20 μl of ABTS (5 mM), 2 μl of HRP (150 U/ml), 10, 60 or 120 μl LOD (50 U/ml), 10 μl l-lactate standard solutions and deionized water. Microplates were 10 s slowly stirred and incubated at 37 °C for 0, 5, 10, 20, 30 or 40 min in a ThermoScientific Multiscan GO microplate reader. For each incubation time, microplates were 10 s slowly stirred before measuring the absorbance at 420 nm. The zero absorbance was set on air. Regardless the l-lactate concentration, the end point of the reaction was reach after 5 min for mixture containing 1.5 or 2.5 U/ml of LOD, while 20 min were required with 0.25 U/ml of LOD (Fig. 1A). For incubation time higher than 20 min and for the three LOD concentrations, the regression lines are superimposed and showed good determination coefficient, above 0.99 (Fig. 1B). In a perspective of high throughput quantification of l-lactate, a 1.5 U/ml loading of LOD was chosen for the next steps.

### Determination of the lower limits of detection and quantification

To determine the lower limits of detection (LLOD) and quantification (LLOQ), two other sets of standards distributed on nine levels: 0, 5, 10, 20, 30, 40, 50, 60 and 80 mg/l (0.1 ≤ *A*_420 nm_ ≤ 0.9) were analyzed in duplicate in two separate experiments. The parameters of the model (±standard deviation) were evaluated using a linear regression by least-squares method (Microsoft Excel Software). The linear model which related l-lactate concentration ([LA]) to absorbance at 420 nm was:

$$A_{420\;\text{nm}}=a\times [\text{LA}]+b$$

where *a* was the slope, also called sensibility, and *b* the intercept of the straight line. For manual assays, the sensibility was 1.05 ± 0.01 × 10^−2^ l/mg, the intercept of the straight line was 9.00 l/mg with a standard deviation of 0.52 × 10^−2^ l/mg (*s*_b_). The determination coefficient (*R*^2^) was 0.991 and the residual standard deviation was 0.027. The LLOD and LLOQ at 95% confidence level were calculated as follow: LLOD = (*b* + 3*s*_b_)/*a*; and LLOQ = (*b* + 10*s*_b_)/*a*, and found to be equal to 10 and 13.8 mg/l, respectively.

### Automated assays

Automated assays were carried out using a GENESIS Freedom Evo (Tecan) fitted out with an eight needle pipetting and two microplate handling arms, and a Tecan Infinite200 microplate reader respectively driven by Gemini and Magellan software (Tecan). Standards distributed on 8 levels: 0, 10, 15, 30, 45, 60, 75 and 90 mg/l of l-lactate were repeated 12 times on two different plates. The linear model parameters were *a* = 1.10 ± 0.01 × 10^−2^ l/mg, *b* = 8.67 ± 0.66 × 10^−2^ l/mg, *R*^2^ = 0.987 and the residual standard deviation was 0.038. LLOQ and LLOD were equal to 9.6 and 13.8 mg/l, respectively. Therefore, automated results are in good accordance with manual ones. Accuracy and repeatability were further studied by 16 repeated assays on the standard 62.3 mg/l of l-lactate. The mean value obtained was 60.9 ± 0.8 mg/l with low coefficient of variation (1.3%) and error compared with the target value (2.2%), confirming that the method can be trusted for quantitative measurements.

### Practicality and reliability test on fungal culture supernatants

The practicality and reliability of this method were further studied by measuring, in duplicates, the l-lactate concentration in 9 independent culture supernatants of filamentous fungi producing optically pure l-lactate. The results obtained with the microplate method were compared with HPLC method [4]. Briefly, 20 μL of sample were separated with Aminex HPX-87H column (Biorad) using isocratic system at 35 °C, H_2_SO_4_ 0.05 M as mobile phase at 0.6 ml/min. The detection of (dl)-lactic acid was performed by UV detection at 210 nm (Agilent 1100 series HPLC). The culture supernatants were diluted prior to analysis, without any further preparation, to get in the quantification range. The mean difference (*d*) between the results obtained with both methods was 0.03 g/l and the standard deviation (*s*_*d*_) of the mean difference was 0.7 g/l (Table 1). If the difference are normally distributes, the microplate method may be 1.3 g/l above or below (*d* ± 1.96*s*_*d*_) the HPLC method with 95% confidence. To evaluate the accordance of the methods, a Wilkoxon test was carried out and showed that there was no significant difference between the two methods with 99% confidence. These results show that the two methods are in accordance: the trueness of the microplate method is similar to the trueness of the HPLC method. These results also show that there was low interference of the other products secreted by filamentous fungi, such as other organic acids or enzymes, in the microplate method.

## Concluding recommendations

The high throughput method for the screening of l-lactate concentration described here was successfully applied to microbial culture supernatants. A concentration of 1.5 U/ml is recommended for a faster analysis. For a less expensive assay, a LOD load ranging from 0.25 to 1.5 U/ml may be used. A control sample without LOD is recommended to make sure that the color of the supernatant or H_2_O_2_ producing enzymes does not interfere with the reaction. When appropriate, the value of this control should be subtracted from the sample value before the calculations. This colorimetric method could also be combined with miniaturized microbial culture method to achieve a completely automated screening [5]. This would therefore allow the fast analysis of l-lactic acid production from several strains and to optimize the pH, nutrient composition and carbon source that are important for l-lactic acid final yields and costs production.

## Figures and Tables

**Table 1:** Comparison of the automated enzymatic method and the reference HPLC method for l-lactate quantification (g/l).

Supernatant	Colorimetric	HPLC	Difference
1	3.8	3.3	0.5
2	13	12.7	0.3
3	12.3	11.7	0.6
4	2.2	3	−0.8
5	12.7	13.7	−1.0
6	10.6	11.2	−0.6
7	0.9	1.1	−0.2
8	14.8	13.9	0.9
9	3.9	3.9	0.0

*d* (average on difference)			0.03
*s*_*d*_ (standard deviation of difference)			0.7
